# Supplementary material for: Argonaute and Dicer are essential for communication between Trichoderma atroviride and fungal hosts during mycoparasitism
Source: Microbiol Spectr. 2024 Mar 5;12(4):e03165-23. doi: 10.1128/spectrum.03165-23 (PMC10986496; doi:10.1128/spectrum.03165-23)
Supplement: Supplemental figure and tables — Twelve supplemental figures and seven supplemental tables. [file spectrum.03165-23-s0003.pdf]

Argonaute and Dicer are essential for communication between *Trichoderma atroviride* and fungal hosts during mycoparasitism.

Eli Efrain Enriquez-Felix[1], Camilo Pérez-Salazar[1], José Guillermo Rico-Ruiz[1], Ana Calheiros de Carvalho[3], Pablo Cruz-Morales[3], José Manuel Villalobos-Escobedo[2]*, and Alfredo Herrera-Estrella[1]*.

[1]Laboratorio Nacional de Genómica para la Biodiversidad-Unidad de Genómica Avanzada, Cinvestav Campus Guanajuato, Irapuato, 36821, Guanajuato, México.

[2]Department of Plant and Microbial Biology, University of California, Berkeley, CA 94720, United States.

[3]Novo Nordisk Center for Biosustainability, Technical University of Denmark.

***Corresponding Authors.**

Supplementary figures

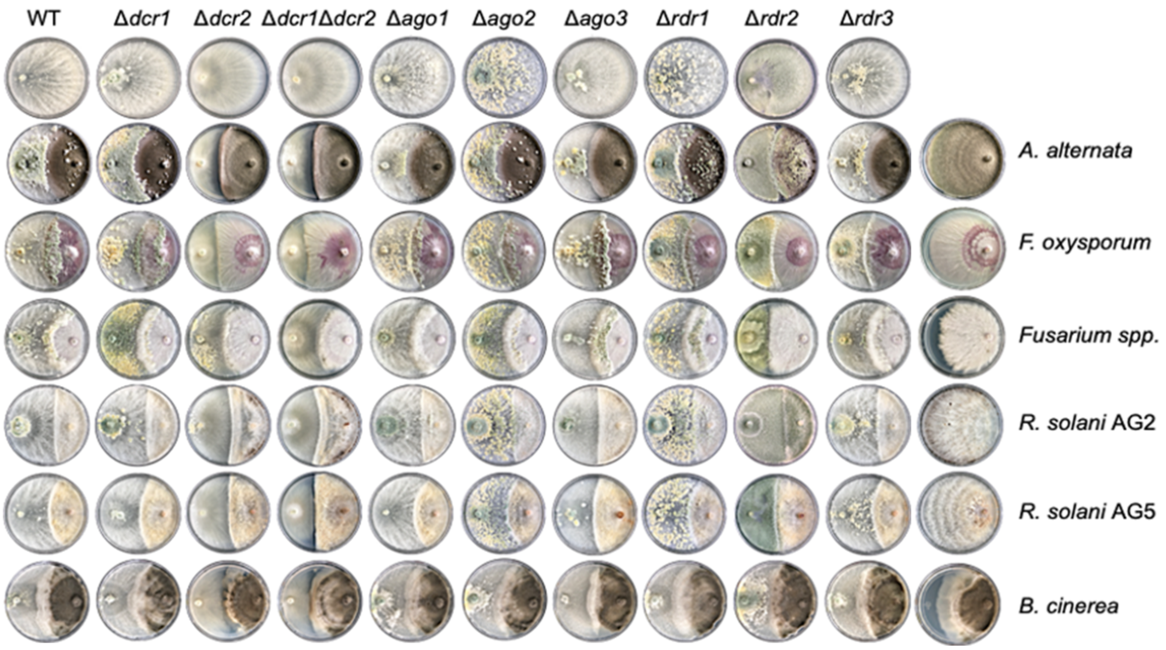

Figure S1. Dual culture assays of RNAi mutant strains against some fungal hosts. The *T. atroviride* strains were inoculated on the left side, and the fungal hosts on the right side. The plates in the top row show the *T. atroviride* strains growing alone, and those in the right-most column represent the fungal hosts growing alone. Mycelium plugs of *T. atroviride* strains were inoculated on the left side of the Petri dish, and the fungal hosts were inoculated on the right side of the plate. The dual cultures were established in PDA at 27°C.

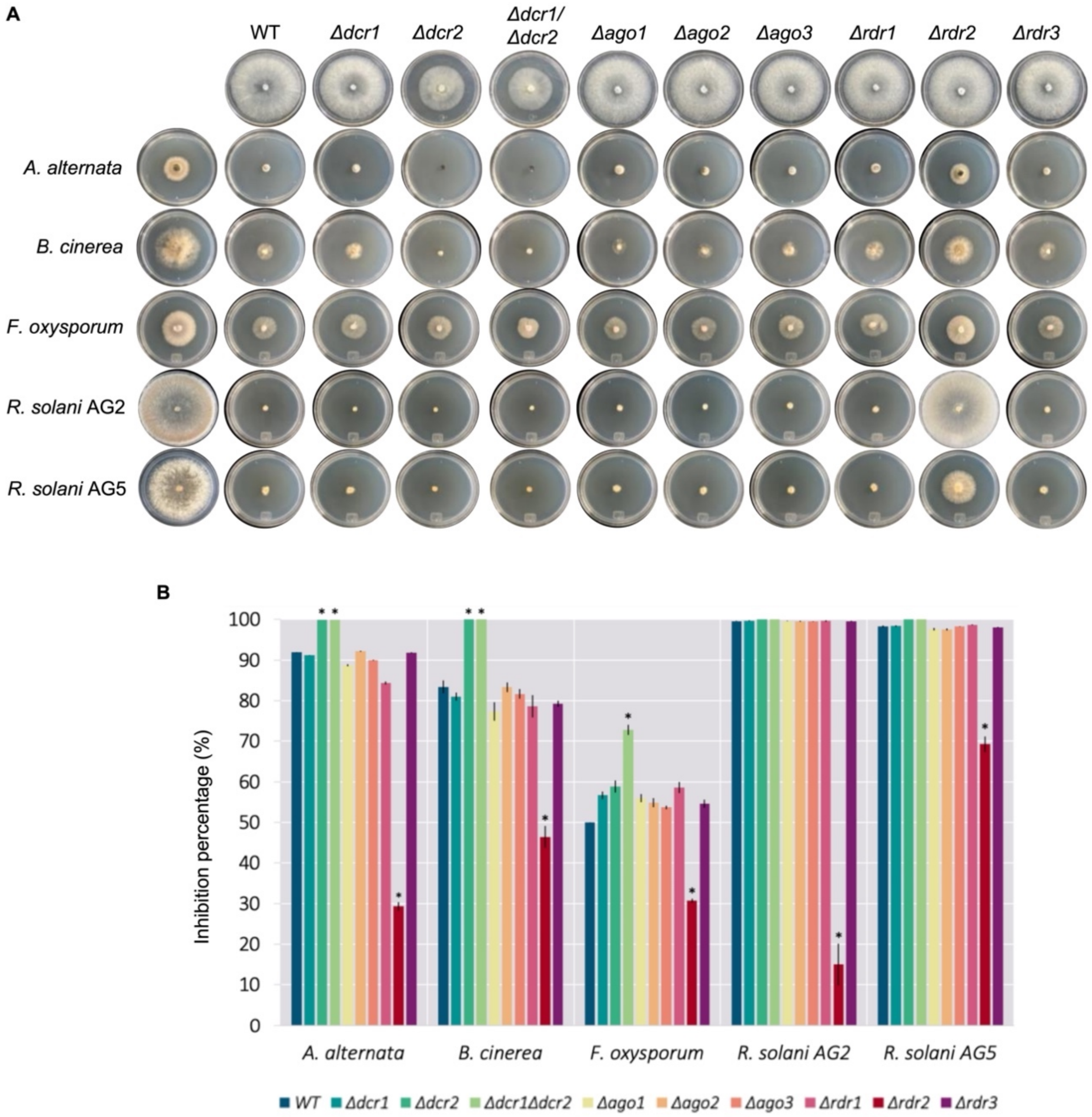

Figure S2. The RNAi machinery controls antibiosis. (A) Representative photographs of plates showing the inhibition of different fungal species by RNAi mutant strains. (B) The graph shows the growth inhibition of the indicated pathogens by the different *Trichoderma* strains (see color code at the bottom). Percentage of inhibition of 3 biological replicas at different times. Each bar represents the average of three biological replicas performed independently. ANOVA one-way and a t-test were performed to compare each mutant against the WT strain (*: $P < 0.05$).

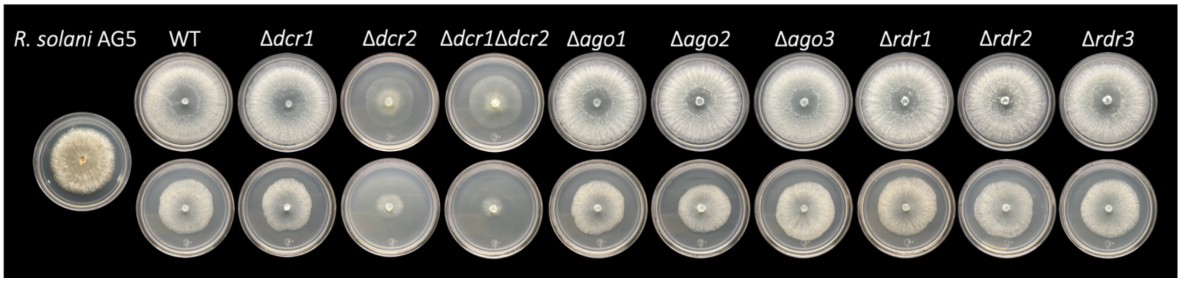

Figure S3. R. solani AG5 diffusible compounds exert growth inhibition on the *T. atroviride* strains. The photographs show the effect of soluble compounds produced by *R. solani* AG5 on the growth of the *T. atroviride* WT and RNAi machinery mutant strains. The top row corresponds to the growth of the *T. atroviride* strains on plates with no diffusible compounds (control). The bottom row shows the growth of the *T. atroviride* strains on plates where *R. solani* AG5 was grown (treatment). Growth of the *R. solani* AG5 strain is shown in the first column.

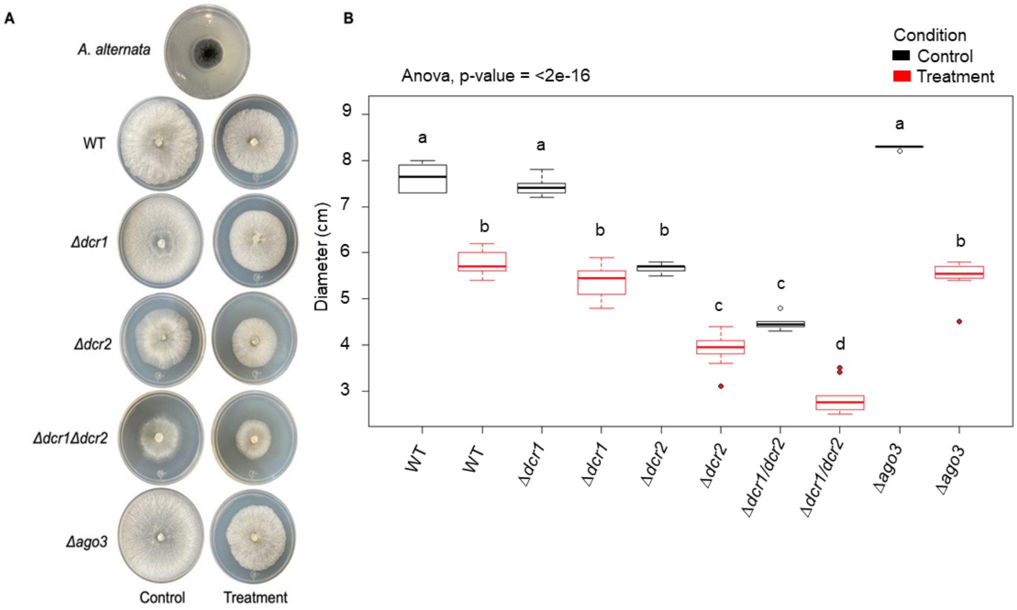

Figure S4. Growth inhibition exerted by *A. alternata* diffusible compounds on the *T. atroviride* strains. (A) The photographs show the effect of soluble compounds produced by *A. alternata* on the growth of the *T. atroviride* WT and RNAi machinery mutant strains. The first column corresponds to the growth of the *T. atroviride* strains on plates without diffusible compounds (control). The second column shows the growth of the *T. atroviride* strains on plates where *A. alternata* was previously grown (treatment). Growth of the *A. alternata* strain is shown at the top. (B) Colony diameter of the *T. atroviride* strains growing in the presence of *A. alternata* diffusible compounds (red bars, treatment) or without them (black bars, control). The values represent the mean of three biological replicas. A one-way ANOVA and a TukeyHSD test were performed to determine statistical differences among the strains in the same treatment ($P < 0.05$).

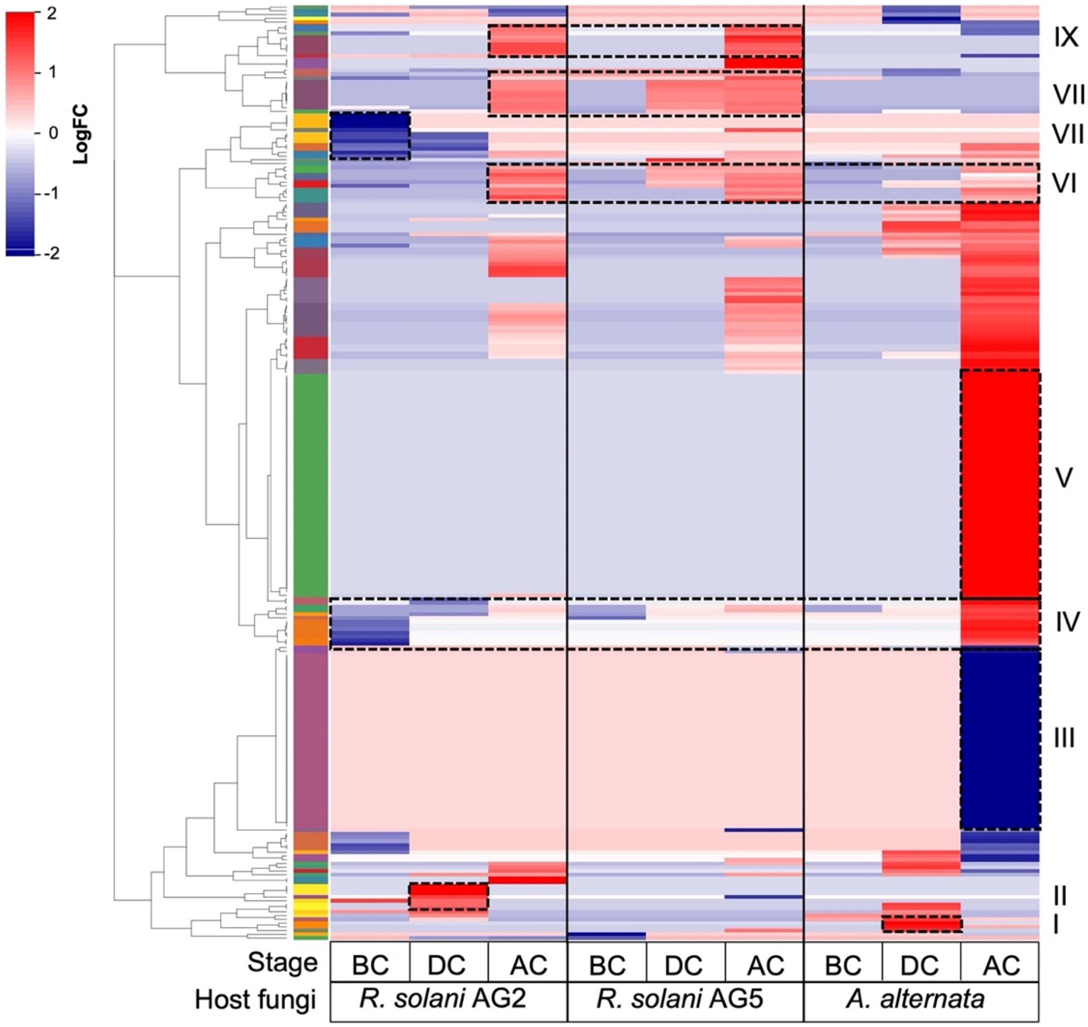

Figure S5. Heatmap of DEGs coding to CAZymes during confrontation of *T. atroviride* WT with *R. solani* AG5/AG2 or *A. alternata*. LogFC of DEGs from WT confrontations compared to the WT control at three stages of mycoparasitism (BC: before contact, DC: during contact, and AC: after contact). The different clusters identified in the dendrogram are represented by the colored bar on the left side of the heatmap. The constitution of the nine clusters of genes indicated in Roman numbers is detailed in Data Set 2.

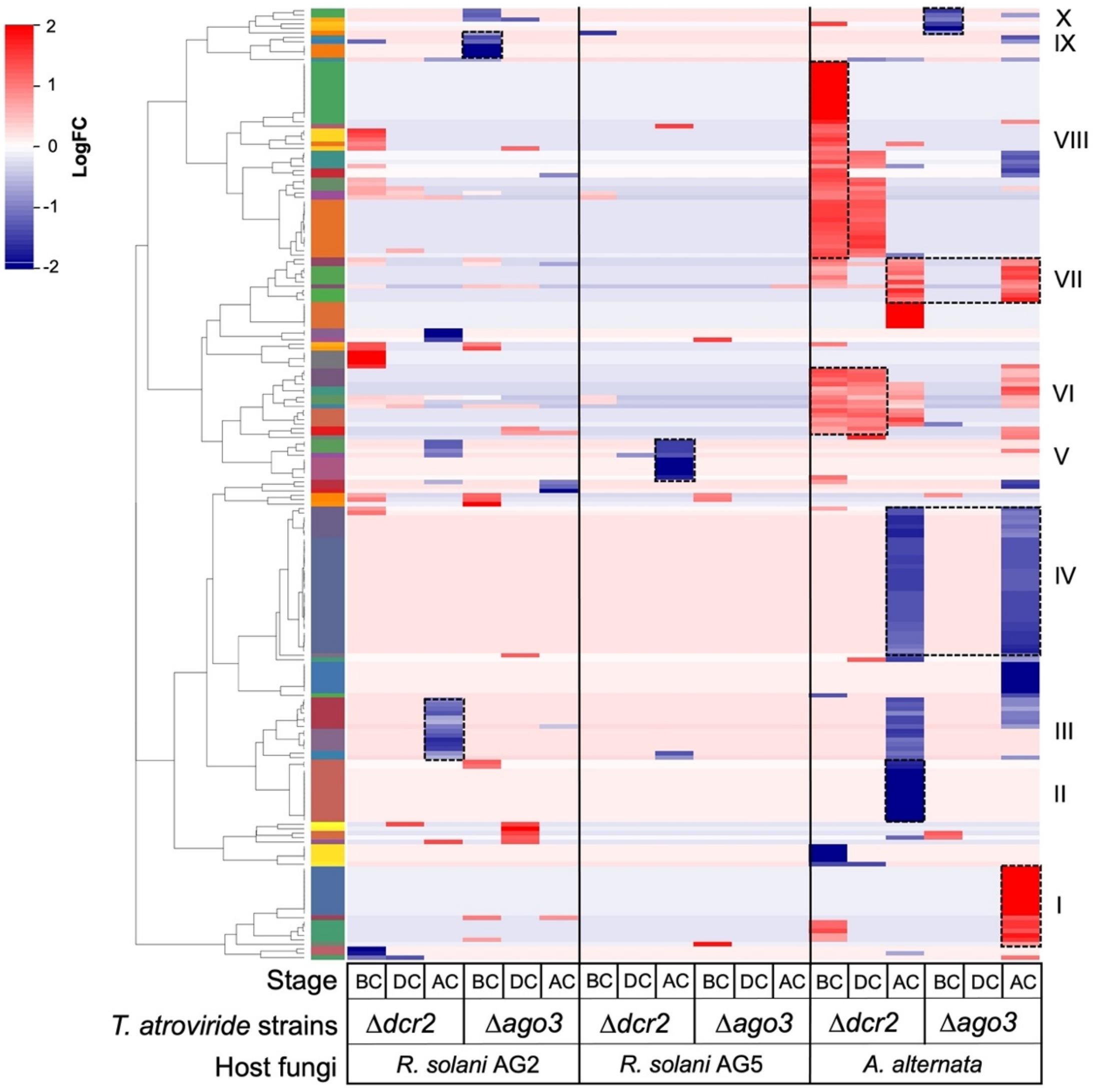

Figure S6. Heatmap showing DEGs coding to Cazymes found in the *Δdcr2* and *Δago3* libraries compared to WT strain. The DEGs coding to glycoside hydrolases (GHs), polysaccharide lyases (PLs), carbohydrate esterases (CEs), glycosyl transferases (GTs), and enzymes with auxiliary activities (AAs) were filtered, and a Pearson correlation was used to cluster the genes. BC: before contact, DC: during contact, and AC: after contact. The different clusters identified in the dendrogram are represented by the colored bar on the left side of the heatmap. The constitution of the nine clusters of genes indicated in Roman numbers is detailed in Data Set 2.

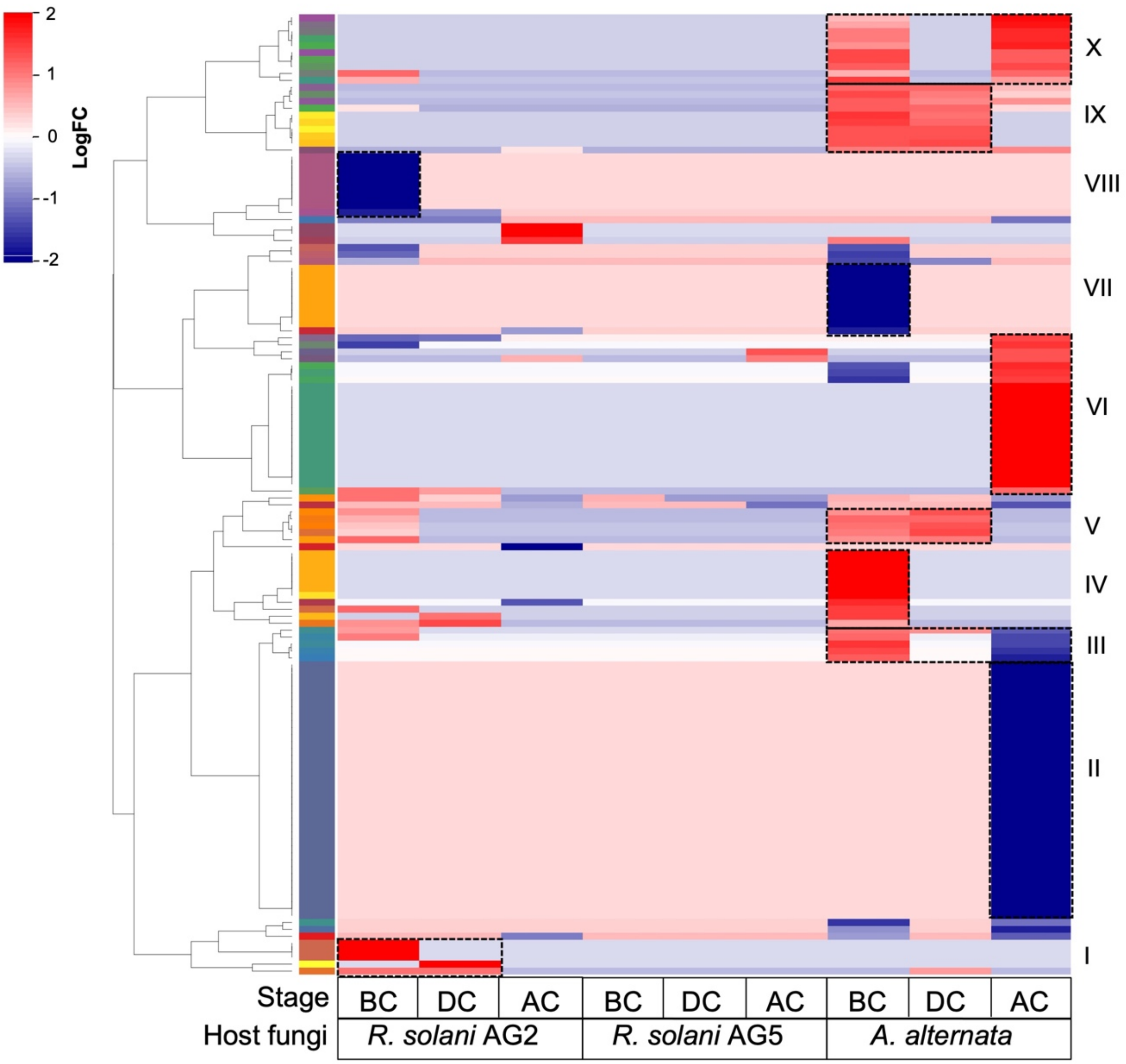

Figure S7. Heatmap showing DEGs coding to MFS and ABC transporters found in *Δdcr2* strain libraries compared to WT strain. The DEGs coding to MFS and ABC transporters in the confrontations *Δdcr2* vs. R. solani AG2, Δdcr2 vs. R. solani AG5, and Δdcr2 vs. *A. alternata* in all stages. BC: before contact, DC: during contact, and AC: after contact. The different clusters identified in the dendrogram are represented by the colored bar on the left side of the heatmap. The constitution of the nine clusters of genes indicated in Roman numbers is detailed in Data Set 2.

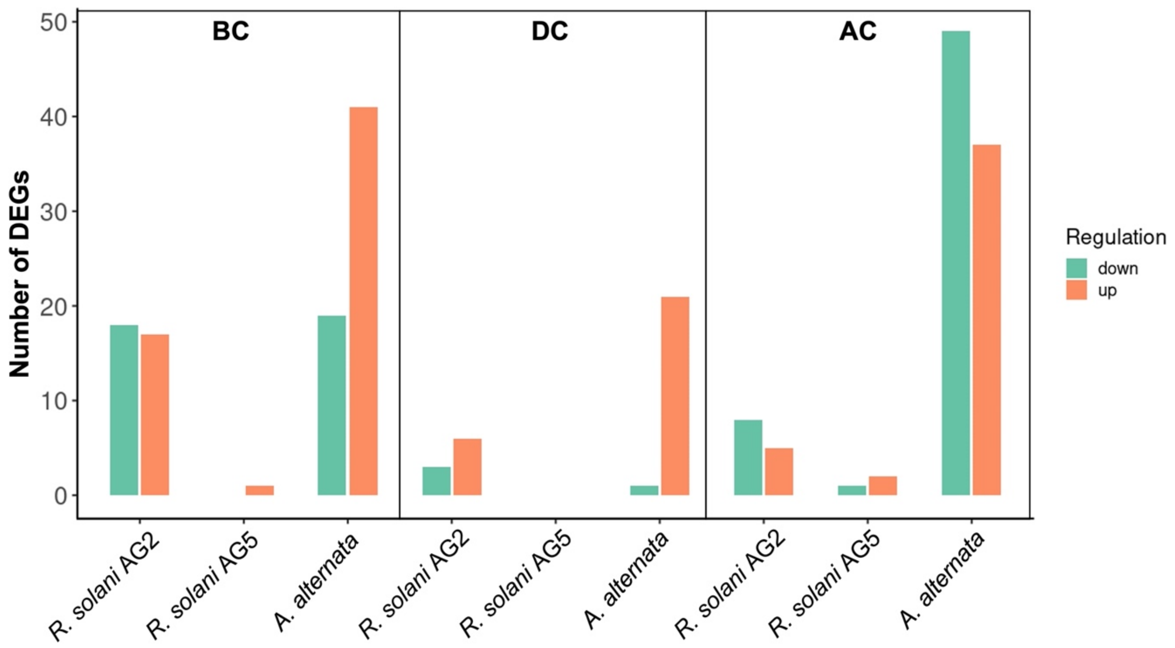

Figure S8. Differentially expressed genes DEGs encoding MFS and ABC transporters in the *Δdcr2*. Number of DEGs encoding transporters in the *Δdcr2* strain in confrontation with different phytopathogenic fungi at different times. **BC**: before contact, **DC**: during contact, and **AC**: after contact.

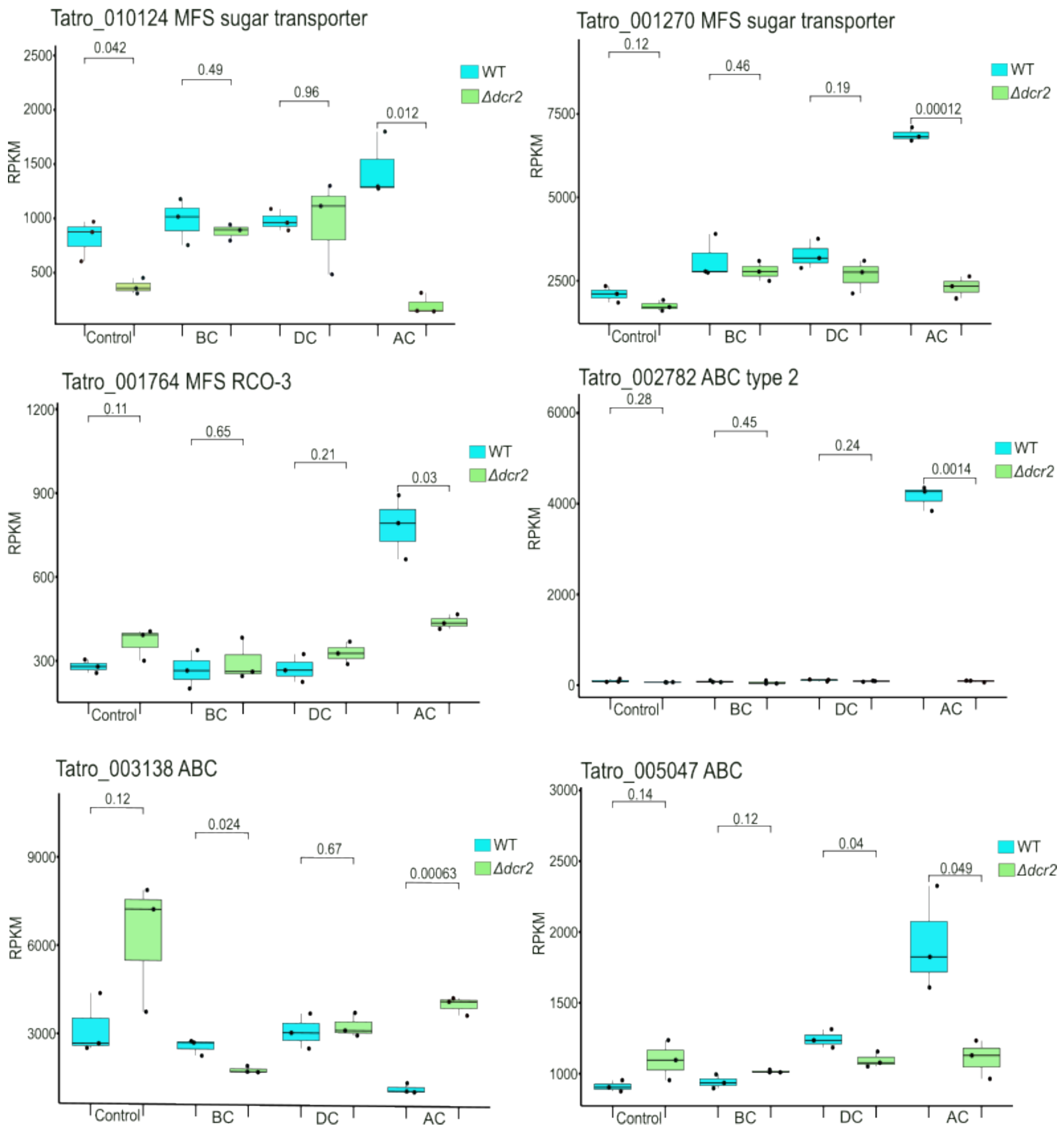

Figure S9. Expression of genes encoding MFS and ABC transporters deregulated in *Δdcr2 vs. A. alternata* in the different confrontations. Boxplots represent the mean of the Reads Per Kilobase of transcript per Million mapped reads (RPKM) of three biological replicas for each gene in the WT and *Δdcr2* libraries. ANOVA one-way and a t-test were performed to compare the means of the *Δdcr2* strain against the WT strain for each stage (the p-value is shown for each comparison). BC: before contact, DC: during contact, and AC: after contact. MFS: Major Facilitator Superfamily transporter. ABC: ATP-binding cassette transporter.

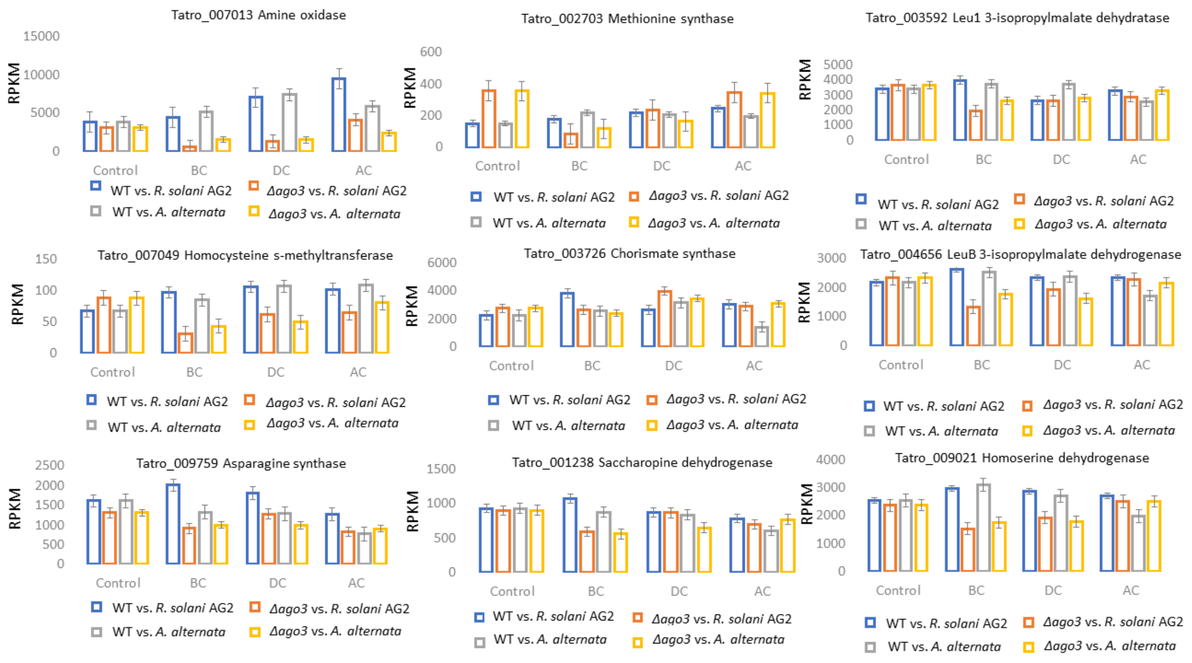

Figure S10. Expression of genes involved in amino acid metabolism in the WT and *Δago*3 libraries. These genes are involved in the biosynthesis of methionine, cysteine, leucine, aromatic amino acids, asparagine, and lysine. The gene expression is more drastically affected at the BC stage in the Δ*ago*3 strain. The X-axis shows the RPKM value for each gene, and the Y-axis indicates the stage of the interaction (BC: before contact, DC: during contact, and AC: after contact).

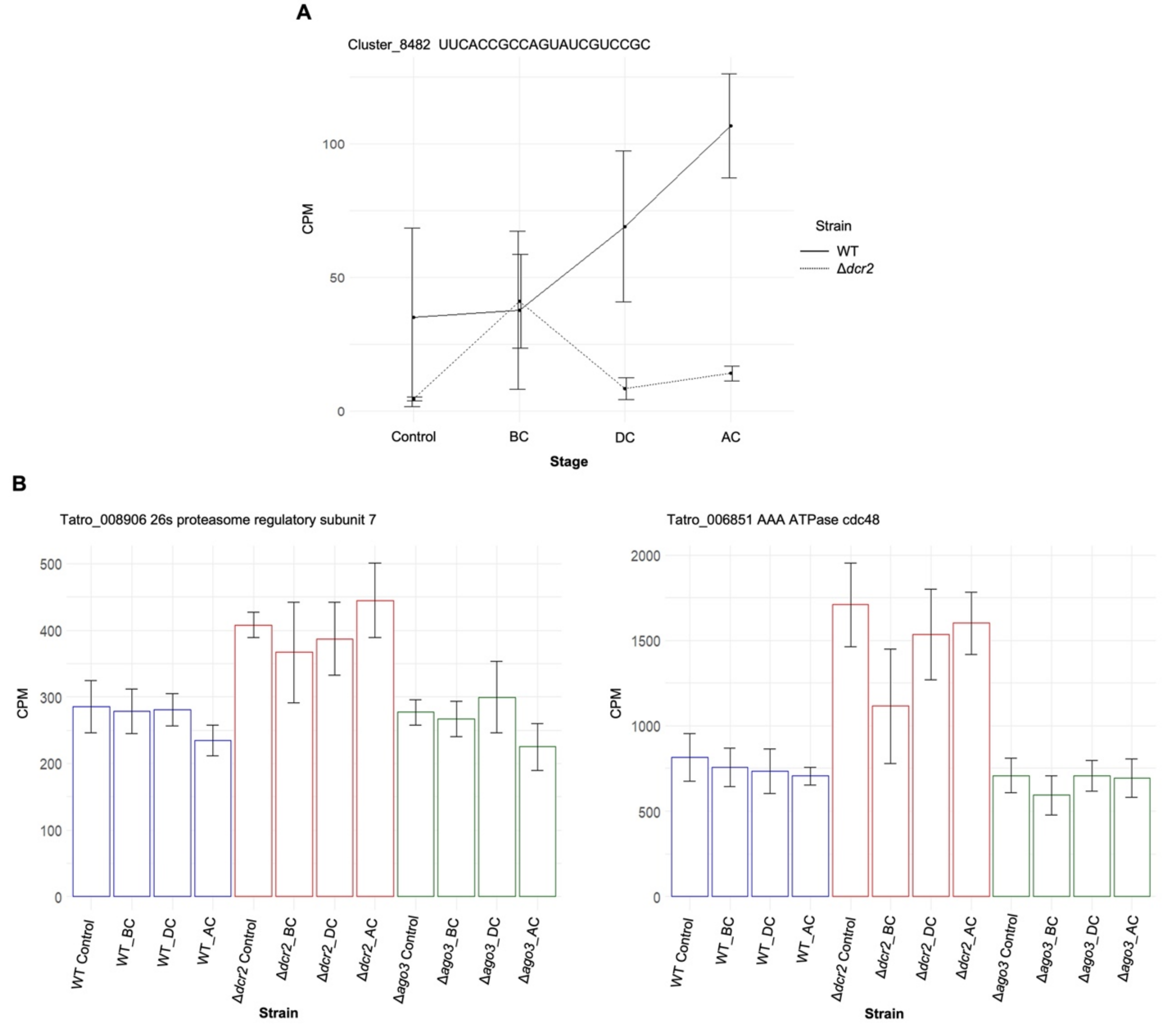

Figure S11. Expression of siRNAs and their targets. Counts per million (CPM) of some up-regulated siRNAs (A) and the expression of their predicted targets (B) in RNA-seq libraries of WT, Δ*dcr*2, and Δago3 strains in different times (BC: before contact, DC: during contact, and AC: after contact). This set of predicted targets were up-regulated in *Δdcr2* compared to the WT in our transcriptomic data. The middle line of each bar represents the standard deviation of three biological replicates.

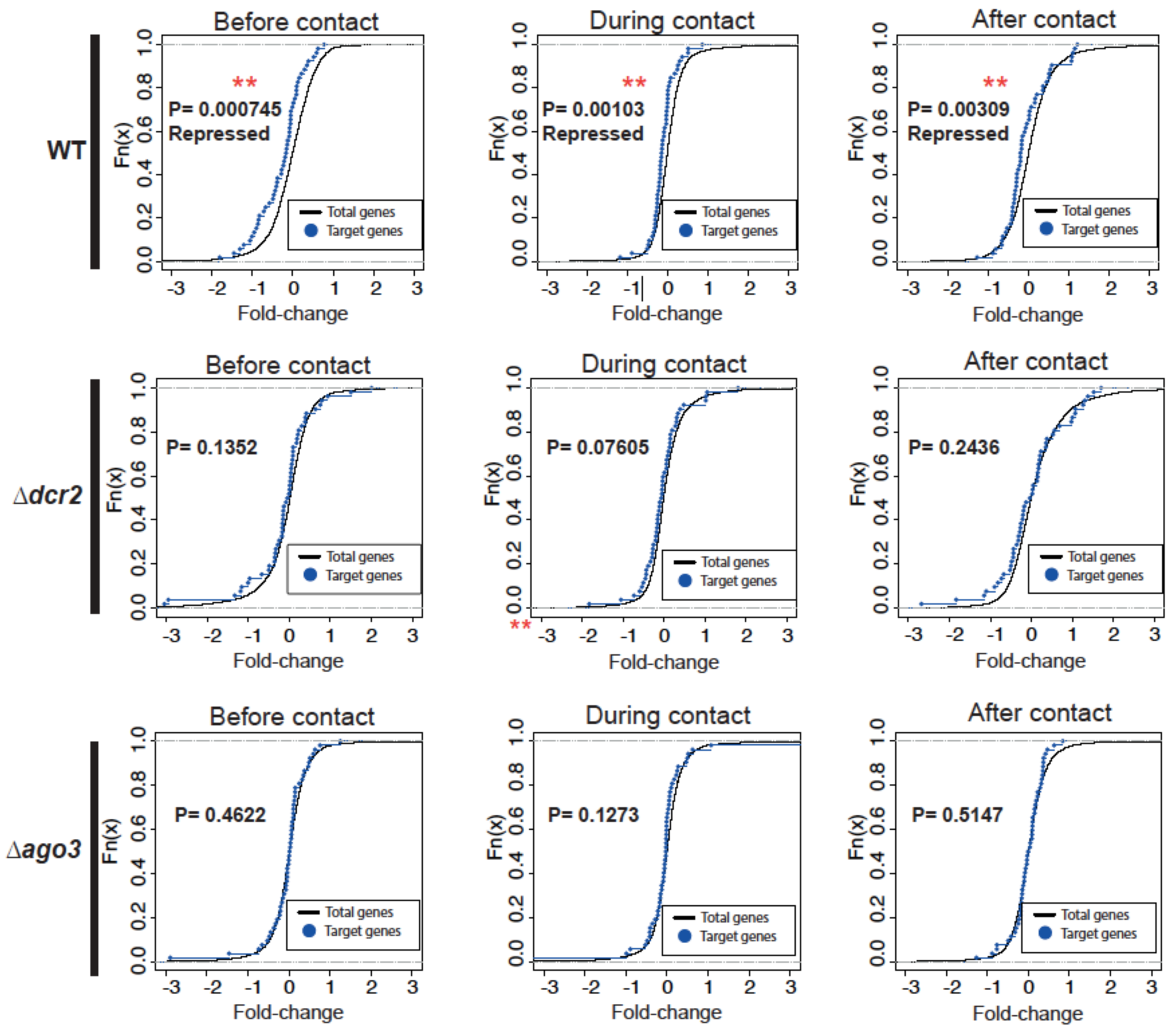

Figure S12. Plots of predicted target genes against the total genes in the transcriptomic libraries. Graphs showing Wilcoxon test results between predicted targets in the WT *vs*. *R. solani* AG5, *Δdcr2 vs. R. solani* AG5 and *Δago3 vs*. *R. solani* AG5 interaction.

Supplemental table 1. Common differentially expressed genes in *Δdcr2* and *Δago3* libraries.

**Up-regulated genes**			
**ID**	**Description**	**logFC**	**PValue**
Tatro_000098	hypothetical protein	2.05	7.31E-10
Tatro_000406	myb family transcription factor	5.11	8.57E-53
Tatro_000633	C2H2 transcription factor	5.07	1.21E-15
Tatro_001844	hypothetical protein	2.47	3.64E-19
Tatro_002011	tripeptidyl-peptidase 1	2.73	8.58E-09
Tatro_002179	Monooxygenase	1.14	0.000146534
Tatro_007162	hypothetical protein	1.63	2.14E-05
Tatro_009148	hypothetical protein	7.46	2.41E-84
Tatro_009263	MFS	1.16	2.61E-05
Tatro_010835	oxidoreductase -like protein	2.32	1.90E-05
Tatro_011181	redoxin domain-containing protein	2.71	1.04E-06
**Down-regulated genes**			
**ID**	**Description**	**logFC**	**PValue**
Tatro_010976	protein iq-domain 14-like	-8.57	3.72442992349576E-08
Tatro_007043	multicopper like protein	-4.77	6.05661753486122E-16
Tatro_000618	short-chain dehydrogenase	-4.53	2.02024693253447E-09
Tatro_004123	Hydrophobin HFBII	-4.52	9.05211016037258E-09
Tatro_011069	cupin domain-containing protein	-3.33	1.58529675730864E-27
Tatro_007091	glycosyltransferase family 8 protein	-3.16	0.000125633431758285
Tatro_000619	cytochrome p450	-3.11	1.45961295105868E-12
Tatro_011257	aminoglycoside phosphotransferase	-2.95	7.42251670507332E-11
Tatro_011070	short chain dehydrogenase reductase	-2.49	9.4469861159997E-16
Tatro_000613	expression library immunization antigen 1	-2.36	3.04344775575274E-11
Tatro_011258	integral membrane protein	-2.35	9.46733201067706E-09
Tatro_000863	acid phosphatase pho84	-2.24	1.16832513711803E-08
Tatro_010376	Superoxide dismutase [Mn], mitochondrial	-2.19	5.29215227419462E-17
Tatro_000896	hypothetical protein	-2.15	0.000119224598505061
Tatro_011081	efflux pump antibiotic resistance	-2.09	5.16062167360858E-07
Tatro_006522	nad dependent epimerase dehydratase	-1.93	5.07174847058605E-07
Tatro_005972	rheb small monomeric gtpase	-1.85	4.56463648812058E-19
Tatro_003825	sh3 domain-containing protein	-1.79	1.85436152778343E-14
Tatro_005635	catechol o-methyltransferase	-1.77	0.000119195755607005
Tatro_003171	meiotic chromosome segregation protein	-1.66	2.10218604912827E-06
Tatro_010363	hypothetical protein	-1.64	1.6585657355703E-06
Tatro_002648	hypothetical protein	-1.47	5.95831388421953E-05
Tatro_003041	ribonuclease p mrp	-1.44	0.000247655162568047
Tatro_007365	hypothetical protein	-1.41	1.13928105581829E-05
Tatro_010767	hexose transport-related protein	-1.31	2.13591967438837E-05
Tatro_007128	f-box domain containing protein	-1.11	0.000199045290072838
Tatro_000404	amino acid permease family	-1.10	8.60172041875525E-06
Tatro_007421	Alternative oxidase, mitochondrial	-1.03	0.000191121592633756

Supplementary table 2. Gene Ontology enrichments for each module in the co-expression network.

Module color	Functional category
Pink	**-Biological process:**<br>Carbohydrate metabolism.<br>**-Molecular function:**<br>DNA binding.<br>Hydrolase activity.<br>Hydrolysis of o-glycosyl compounds.<br>Transport activity.<br>Cellulose binding.
Purple	**-Biological process:**<br>Regulation of transcription DNA-templated.<br>Regulation of nucleobase-containing compound metabolic process.<br>Regulation of RNA biosynthetic process.<br>Regulation of nucleic acid-templated transcription.<br>Cellular response to stimulus.<br>Cell communication.<br>Signaling.
Brown	**-Biological process:**<br>Carbohydrate metabolic process.<br>Proteolysis.<br>Ion transmembrane transport.<br>Amino acid transport.<br>Anion transmembrane transport.<br>Organic acid transport.<br>Amino acid transmembrane transport.<br>Organic acid transmembrane transport.<br>Carboxylic acid transmembrane transport.
Blue	**-Biological process:**<br>Phosphorus metabolic process.<br>Lipid metabolic process.<br>Cell communication.<br>Signal transduction.<br>Signaling.<br>Lipid biosynthetic process.<br>Intracellular signal transduction.<br>Small GTPase mediated signal transduction.
Yellow	**-Biological process:**<br>Organonitrogen compound biosynthetic process.<br>Cellular protein metabolic process.<br>Translation.<br>Peptide biosynthetic process.<br>Amide biosynthetic process.<br>Peptide metabolic process.<br>Cellular amide metabolic process.<br>Cellular component biogenesis.<br>Alpha-amino acid metabolic process.<br>Protein-containing complex subunit organization.

Green	**-Biological process:**<br>RNA processing.<br>Cellular component.<br>Ribosome biogenesis.<br>ncRNA, rRNA, mRNA, tRNA processing.
Turquoise	**-Biological process:**<br>Organic acid metabolic process.<br>Catabolic process.<br>Organic substance catabolic process.<br>Cellular catabolic process.<br>Organonitrogen compound catabolic process.<br>Protein catabolic process.<br>Cellular macromolecule catabolic process.<br>tRNA metabolic process.<br>Cellular protein catabolic process.

Supplemental table 3. Hub genes for each gene co-expression network module.

**Module**	**Hub gene ID**	**Funtion**
Turquoise	Tatro_011105	Regulator of chromosome condensation-like protein
Green	Tatro_007468	Peptidyl-prolyl type FKBP
Yellow	Tatro_005063	60s ribosomal protein L27
Blue	Tatro_007409	B-glucosidase SUN4 septation protein
Purple	Tatro_005473	Ste12-like proteins
Brown	Tatro_010117	Major Facilitator Superfamily (MFS) ion channel
Pink	Tatro_008072	Exo-glucanase CBH2

Supplemental table 4. Genes with high connectivity for brown, blue, and turquoise modules.

Module	ID gene	Funtion
Brown	Tatro_002148	G-protein coupled receptor
	Tatro_007149	DUF323 domain protein
	Tatro_000312	DUF614 domain protein
	Tatro_011504	Cysteine protease
	Tatro_009532	Cytochrome-NADH B5
	Tatro_010400	YTP1 protein
	Tatro_003847	N-acetyltransferase (GNAT family)
	Tatro_009912	Protein with glutaredoxin domain
	Tatro_010905	Polyketide synthase
Blue	Tatro_008206	Iron permease Fam. FTR1
	Tatro_000030	NADPH oxidase
	Tatro_006239	WSC domain protein
	Tatro_004945	PRO41 protein
	Tatro_006936	C6 transcription factor
	Tatro_003931	SUN domain protein
	Tatro_003873	PRP4 domain protein
	Tatro_001849	ECM33-like protein
	Tatro_001627	Major facilitator superfamily (MFS)
	Tatro_008703	WSC domain protein
	Tatro_010796	endoglucanase GH45
	Tatro_009048	β-1,3(4)-endoglucanase GH16
	Tatro_005853	endo-β-1,4-glucanase GH3
	Tatro_010324	endo-β-1,4-glucanase GH7
	Tatro_001209	EBP domain protein
Turquoise	Tatro_003784	RNA polymerase
	Tatro_011164	HET domain protein
	Tatro_004392	Vacuolar ATP synthase
	Tatro_006998	Polysaccharide synthase
	Tatro_011630	Family protein AIG2
	Tatro_011247	MYND-like zinc domain protein
	Tatro_002695	nitrogen regulator
	Tatro_009061	NUDIX family proteins
	Tatro_009640	DEAD/DEAH-box helicase
	Tatro_001332	Ubiquitin ligase E3
	Tatro_008538	ADP-ribosylglycohydrolase
	Tatro_006226	Major facilitator superfamily (MFS)

Supplemental table 5. Predicted milRNA in *T. atroviride* using ShortStack and mirDeep2.

milRNA (ID)	mature milRNA sequence (5' - 3')	Strand	Locus	Shared*
**Tatro_milR- 1**	UAUCAGAAUUCGUUGGGUAGC	-	contig_1:2047613-2047679	Not shared
**Tatro_milR-2**	UGAAACCCCGGACAAACUUGC	-	contig_1:3319055-3319161	*T. asperellum*
**Tatro_milR- 3**	GGUCUGGUGGUCUAGUGG	+	contig_1:4787874-4787914	*T. reesei, T. gamsii*
**Tatro_milR- 4**	CGAGCGCAAGGCUGCCGGCCGUG	-	contig_1:5669093-5669143	*T. asperellum, T. gamsii, T. virens, T. simmonsii, T. reesei, T. citrinoviride, T. pseudokoningii*
**Tatro_milR- 5**	ACCCUAAUCUUGCGUCUCUGGCCC	-	contig_1:5850570-5850642	*T. asperellum, T. virens*
**Tatro_milR- 6**	AAGGAGAACGCCGUCAAGGGC	+	contig_1:6017442-6017522	*T. citrinoviride, T. asperellum, T. gamsii, T. pseudokoningii, T. simmonsii, T. reesei, T. harzianum, T. virens*
**Tatro_milR-7**	UUCCAUUAUUUAUUCAAAGACG	+	contig_2:2739573-2739605	Not shared
**Tatro_milR-8**	AGCGGAAGGGUUGGGAGUGGUG	-	contig_2:4081693-4081814	Not shared
**Tatro_milR-9**	UCGCGAAGUCGAAAGUGACAGC	-	contig_2:4233523-4233736	Not shared
**Tatro_milR- 10**	UCUGAAGGUCGUGUGUUC	-	contig_2:4285670-4285709	*T. asperellum, T. simmonsii, T. reesei, T. pseudokoningii, T. virens, Beauveria bassiana*
**Tatro_milR- 11**	GCGACACUGCUGCUCUAGCAACC	+	contig_2:4571020-4571093	Not shared
**Tatro_milR- 12**	UGUCCCUUGCAGGCUUCGGC	+	contig_2:5582204-5582282	*T. reesei, T. virens, T. asperellum, T. simmonsii*
**Tatro_milR- 13**	GCUCCAGCGACAGAACCCU	+	contig_2:5928713-5928751	Not shared
**Tatro_milR-14**	UAAACUACCUAUUAUCGUGACU	+	contig_3:3263813-3263853	Not shared
**Tatro_milR-15**	UAUGUAGAUUGAUCUUCCUCAGA	+	contig_4:1779193-1779331	Not shared

**Tatro_milR- 16**	CAGCUCUAAUGCCCAUACCCU	-	contig_4:2713317-2713370	*T. asperellum*
**Tatro_milR-17**	CUGCUGGAUAACUUUCCCCAAA	+	contig_4:3681699-3681874	Not shared
**Tatro_milR-18**	UGUUAGUAGAGGUAUUGUGUCU	+	contig_5:564030-564068	Not shared
**Tatro_milR-19**	UUAGUAGAGGUAUUGUGUCUA	+	contig_5:564046-564094	Not shared
**Tatro_milR- 20**	GGAGGAGUCGAAGGUGUAGUGCACU	-	contig_5:850369-850424	Not shared
**Tatro_milR-21**	UUUUGCGAUGCCCAAUAUCUGU	+	contig_5:1130859-1130987	Not shared
**Tatro_milR- 22**	UGAAAGCGCGGUUAUCUGUAGC	-	contig_5:1130629-1130717	Not shared
**Tatro_milR- 23**	CCGCGAUGGUCUCGCUGGCGGCGA	-	contig_5:2281288-2281379	*T. asperellum*
**Tatro_milR- 24**	AGCCGGUACUGAGAAGGGAGC	+	contig_6:422694-422746	*T. asperellum*
**Tatro_milR- 25**	UCACCGUUUGACCAUUACAGC	+	contig_6:2660193-2660280	*T. asperellum*
**Tatro_milR- 26**	UAAAUUGAGGGUCUGCAACUCA	+	contig_7:602188-602279	Not shared
**Tatro_milR-27**	UUCGGUCUCGAUGACGUUGACU	+	contig_7:908869-908954	Not shared
**Tatro_milR-28**	UAGCCAGUGCUGUAGCAGACCAUC	+	contig_9:973865-974020	Not shared
**Tatro_milR- 29**	UGACUACUAAUCUGCCGGUUA	-	contig_10:67688-67791	*T. asperellum, T. pseudokoningii, T. simmonsii, T. reesei, T. harzianum, T. citrinoviride*
**Tatro_milR- 30**	UCCUGAGACCGGUAGACUGCA	-	contig_10:1042911-1042953	*T. asperellum, T. gamsii*

Supplemental table 6. Number of small RNA clusters loci ranging from 20 to 26 nucleotides obtained by ShortStack.

Condition	Control				Before Contact				During Contact				After contact			
Replica	R1	R2	R3	Mean	R1	R2	R3	Mean	R1	R2	R3	Mean	R1	R2	R3	Mean
WT	1928	645	3737	2103,33	1057	155	1039	750,33	630	169	1641	813,33	891	207	1315	804,33
Δdcr2	45	30	33	36	21	3	84	36	100	116	378	198	24	104	ND	64

Supplemental table 7. Wilcoxon test between the expression of siRNA targets in the WT libraries versus the corresponding expression in Δ*dcr2* and Δ*ago3* libraries.

**Contrast**	**P-value**	**Regulation**	**Contrast**	**P-value**	**Regulation**	**Contrast**	**P-value**	**Regulation**
WT *vs*. A. *alternata* BC	0.5978	-	Δ*ago3* vs. A. *alternata* BC	0.669	-	*Δdcr2 vs*. A. a*lternata* BC	0.6582	-
WT *vs*. A. *alternata* DC	0.0008037	Repressed	Δ*ago3* vs. A. *alternata* DC	0.1679	-	*Δdcr2 vs*. A. a*lternata* DC	3.06E-02	Repressed
WT *vs*. *A. alternata* AC	0.03855	Repressed	Δ*ago3 vs*. A. *alternata* AC	0.2264	-	*Δdcr2 vs*. A. a*lternata* AC	0.7251	-
WT *vs*. *R. solani* AG2 BC	0.1849	-	Δ*ago3 vs*. *R. solani* AG2 BC	0.1091	-	*Δdcr2 vs*. *R. solani* AG2 BC	0.08411	-
WT *vs*. *R. solani* AG2 DC	0.05406	-	Δ*ago3 vs*. *R. solani* AG2 DC	0.1748	-	*Δdcr2 vs*. *R. solani* AG2 DC	0.3723	-
WT *vs*. *R. solani* AG2 AC	0.03409	Repressed	Δ*ago3 vs*. *R. solani* AG2 AC	0.1079	-	*Δdcr2 vs*. *R. solani* AG2 AC	0.4072	-
WT *vs*. *R. solani* AG5 BC	3.09E-02	Repressed	Δ*ago3 vs*. *R. solani* AG5 BC	0.2436	-	*Δdcr2 vs*. *R. solani* AG5 BC	0.5147	-
WT *vs*. *R. solani* AG5 DC	0.0007455	Repressed	Δ*ago3 vs*. *R. solani* AG5 DC	0.1352	-	*Δdcr2* vs. *R. solani* AG5 DC	0.4622	-
WT *vs*. *R. solani* AG5 AC	0.00103	Repressed	Δ*ago3 vs*. *R. solani* AG5 AC	0.07605	-	*Δdcr2 vs*. *R. solani* AG5 AC	0.1273	-

BC: before contact, DC: during contact and AC: after contact.
